# On the Beneficial Effects Resulting from Mercurial Inunctions in Typhus, Illustrated by the Case of a Private in the 21st Regiment of Dragoons

**Published:** 1804-07-01

**Authors:** 

**Affiliations:** Romsey


*On the beneficial Effects resulting from Mercurial Inunctions in Typhus, illustrated by the Case of a Private in the 21st Regiment of Dragoons, attended by* Mr. Cuming, *in Romsey.*

In the statement of this case, it shall not be so much my business to enter into the minutiæ of detail, as to give you the outlines of the patient's symptoms and my practice tice; for, in general, the monotonous recital of a diary is extremely dry and irksome.

On the 19th of May, I was desired to visit —— Benson, who I found in a state of pyrexia, accompanied with dysentery; his dejections were frequent and bloody, and his countenance remarkably sallow. I prescribed gr. x. pulv. ipecac. comp. 3tia. q. h. sumend. but finding the opium contained in this medicine, insufficient to procure him repose, he took tinct. opii gtt. 40 horâ decubitûs. In the morning of the 20th, I found him rather better; he had been less disturbed during the night than he had been for some time. It is necessary to remark, that he had been long subject to the complaint, and that now it might properly be denominated chronic dysentery. These medicines were continued, and with the happiest effects, in as much as related to the alvine discharge, which began to assume a natural appearance, without the smallest mixture of blood; though there was not any remission of fever, which from the first belonged to the typhoid type, his pulse being often weak and tremulous, tongue nearly covered with a brown fur, the sensorium greatly affected, from the state of amentia I generally observed him to labour under. On the 21st I applied a blister between his shoulders, which roused him a little, and on the 22d I prescribed the volatile alkali, with tinct. lavend. comp. and a bolus at bed time c. pulv. ipec. comp. gr. x. These remedies were continued without any apparent benefit until the 28th. The corona capitis had been shaved, and cold applications of vinegar and water were constantly used, but did not operate very powerfully in restoring lost energy, yet I am persuaded they were useful. I also had the patient taken out of bed, and tried the effect of cold affusions, which I believe would have been attended with considerable benefit, if they had been persevered in; but he was so circumstanced, in point of lodging, as to render this plan extremely inconvenient. I therefore had recourse to mercurial inunctions, and by way of exciting their effects more speedily, administered calomel gr. ij. pulv. antimonial. gr. vj. opii gr. ij. cons. ros. q. s. ft. bol. No. iij. capt. j. ter de die.

These remedies were continued till the 6th of June before their action on the system became evident; that is to say, prior to the mouth being affected, or any degree of ptyalism produced, though a surprising amendment was observable; the secretions were set to work; he had for two or three nights been bathed in perspiration, his tongue

 became became moist, and the digestive faculties of the stomach were again exerted; hitherto he had loathed every thing offered to him, save toast and water, rice and rice-milk, Port wine, &c. and these things were not often relished; but now he expressed a wish for animal food, and was allowed beef, mutton, or veal soup. The mercurial applications were discontinued, and the acid vitriol. dilut. with tinct. lavend. com. was given during the day, with an anodyne at night. Many may suppose that some more powerful tonic, such as the decoct. cinchona, would have been preferable; but experience has taught me, that the recovery of men in similar circumstances, has been often retarded by bracing up the system too suddenly. He took at this period near a pint of Port wine in the day, and a tumbler or two of mild ale, which he also had previous and subsequent to the mercurial course. His convalescence was sufficiently rapid to authorize me to conclude that it was quite unnecessary to alter this treatment; the troop marched on the 13th of June, and three or four days prior to this time his wine was gradually diminished, and his allowance of ale augmented.

The good effects of the mercury in this case was strikingly displayed; the fever, with every concomitant and untoward symptom, after the dysenteric complaint vanished, continued without intermission, and death seemed already brandishing his dart over his devoted victim, until the paralized organs were roused from their lethiferous lethargy by the stimulus of this potent medicine, which has long been highly extolled in the cure of fevers incident to tropical climes. Then, as there is reason to imagine that almost all fevers are governed by the same laws, though differently modified, according to constitution or country, it is highly probable that a more general adoption of the use of this remedy, in private practice, would be productive of the best effects, and considered a most useful auxiliary in the treatment of complaints that not unfrequently baffle all human skill.

When opportunity offers, it is my intention to transmit you an account of the great and inestimable advantages, to be derived from the liberal use of tinct. digitalis, in cases of pneumonia; when carrying bleeding to such lengths as is generally done, frequently undermines the constitution, and produces dropsies, with other evils of no less magnitude than those which it was designed to cure; for that practice, surely, can never be too much decried, which removes one disease, to make way for another more lingering, and equally fatal.

*June* 15, 1804.

